# Supplementary material for: Mitochondrial genomes and phylogeny of Atratus and Educator Group species of the Melanoconion Section of Culex (Melanoconion) (Diptera: Culicidae)
Source: Mem Inst Oswaldo Cruz. 2026 Mar 2;121:e250125. doi: 10.1590/0074-02760250125 (PMC12952796; doi:10.1590/0074-02760250125)
Supplement: Supplementary material [file 1678-8060-mioc-121-e250125-s.pdf]

TABLE I

Information about specimens used in this study, as species, gender, and localities where field collections were conducted

ID_sequence	ID_collection_code	Species (Morphological identification)	Gender	Country	State	Municipality
PQ389	AM-ITACOATIARA	*Cx.* (*Mel.*) *theobaldi*	male	Brazil	AM	Itacoatiara
PQ390	MG39-118	*Cx.* (*Mel.*) *longistriatus*	male	Brazil	MG	Oliveira
PQ391	Coleta 7 AM- HumaitaZet	*Cx.* (*Mel.*) *zeteki*	female	Brazil	AM	Humaitá
PQ392	MG47-14	*Cx.* (*Mel.*) *zeteki*	male	Brazil	MG	Carmo da Mata
PQ393	MG12-135	*Cx.* (*Mel.*) *longistriatus*	male	Brazil	MG	Coronel Pacheco
PQ394	TO1-1	*Cx.* (*Mel.*) near *vaxus*	male	Brazil	TO	Lagoa da Confusão
PQ395	PI4-100	*Cx.* (*Mel.*) near *commevynensis*	male	Brazil	PI	Ribeiro Gonçalves
PQ396	AC66-I	*Cx.* (*Mel.*) *eknomios*	male	Brazil	AC	Acrelândia
PQ397	SP184-01	*Cx.* (*Mel.*) *trigeminatus*	male	Brazil	SP	Pariquera-Açu
PQ398	AC64-I	*Cx.* (*Mel.*) *eknomios*	male	Brazil	AC	Acrelândia
PQ399	SP184-07	*Cx.* (*Mel.*) *trigeminatus*	male	Brazil	SP	Pariquera-Açu
PQ400	SP157-104	*Cx.* (*Mel.*) *trigeminatus*	male	Brazil	SP	Pariquera-Açu
PQ401	IG20-5	*Cx.* (*Mel.*) *angularis*	male	Brazil	SP	Iguape
PQ402	SP31-14	*Cx.* (*Mel.*) near *vaxus*	male	Brazil	SP	Inúbia Paulista
PQ403	IG20-6	*Cx.* (*Mel.*) *angularis*	male	Brazil	SP	Iguape
PQ404	TO1-9	*Cx.* (*Mel.*) *longistriatus*	male	Brazil	TO	Lagoa da Confusão
PQ405	SP171-111	*Cx.* (*Mel.*) *ensiformis*	male	Brazil	SP	Cananéia
PQ406	MS06-100	*Cx.* (*Mel.*) *theobaldi*	male	Brazil	MS	Aquidauana
PQ407	ES18-109	*Cx.* (*Mel.*) *ensiformis*	male	Brazil	ES	Linhares
PQ408	SP-Ciduni-02	*Cx.* (*Mel.*) *angularis*	male	Brazil	SP	São Paulo
PQ409	SP68-35	*Cx.* (*Mel.*) *dunni*	male	Brazil	SP	Dourado
PQ410	AC30-I	*Cx.* (*Mel.*) *theobaldi*	male	Brazil	AC	Acrelândia
PQ411	MG08-102	*Cx.* (*Mel.*) *dunni*	male	Brazil	MG	Planura

CC BY

TABLE II

Information about on number paired-end reads, assembly reads and mitochondrial sequence length of each specimen

ID_sequence	ID_collection_code	Species (Morphological identification)	Reads 1	Reads 2	Assembled reads	Assembled reads (%)	Not assembled reads	Sequence length (bp)
PQ389	AM-ITACOATIARA	*Cx.* (*Mel.*) *theobaldi*	32302	32302	51950	80,41	12654	11815
PQ390	MG39-118	*Cx.* (*Mel.*) *longistriatus*	62425	62425	92978	74,47	31872	11805
PQ391	Coleta 7 AM- HumaitaZet	*Cx.* (*Mel.*) *zeteki*	60441	60441	114032	94,33	6850	11819
PQ392	MG47-14	*Cx.* (*Mel.*) *zeteki*	45998	45998	90641	98,53	1355	11819
PQ393	MG12-135	*Cx.* (*Mel.*) *longistriatus*	57272	57272	101352	88,48	13192	11808
PQ394	TO1-1	*Cx.* (*Mel.*) near *vaxus*	47331	47331	90670	95,78	3992	11808
PQ395	PI4-100	*Cx.* (*Mel.*) near *commevynensis*	50629	50629	98719	97,49	2539	11810
PQ396	AC66-I	*Cx.* (*Mel.*) *eknomios*	58595	58595	112167	95,71	5023	11805
PQ397	SP184-01	*Cx.* (*Mel.*) *trigeminatus*	50159	50159	94600	94,30	5718	11805
PQ398	AC64-I	*Cx.* (*Mel.*) *eknomios*	68173	68173	120671	88,50	15675	11804
PQ399	SP184-07	*Cx.* (*Mel.*) *trigeminatus*	60159	60159	115559	96,04	4759	11806
PQ400	SP157-104	*Cx.* (*Mel.*) *trigeminatus*	32609	32609	44077	67,58	21141	11807
PQ401	IG20-5	*Cx.* (*Mel.*) *angularis*	56464	56464	110439	97,80	2489	11801
PQ402	SP31-14	*Cx.* (*Mel.*) near *vaxus*	66307	66307	130199	98,18	2415	11806
PQ403	IG20-6	*Cx.* (*Mel.*) *angularis*	74724	74724	144891	96,95	4557	11801
PQ404	TO1-9	*Cx.* (*Mel.*) *longistriatus*	72498	72498	140965	97,22	4031	11806
PQ405	SP171-111	*Cx.* (*Mel.*) *ensiformis*	65606	65606	122565	93,41	8647	11810
PQ406	MS06-100	*Cx.* (*Mel.*) *theobaldi*	55074	55074	106914	97,06	110148	11816
PQ407	ES18-109	*Cx.* (*Mel.*) *ensiformis*	65416	65416	126108	96,39	4724	11811
PQ408	SP-Ciduni-02	*Cx.* (*Mel.*) *angularis*	55658	55658	109805	98,64	1511	11801
PQ409	SP68-35	*Cx.* (*Mel.*) *dunni*	79590	79590	149703	94,05	9477	11807
PQ410	AC30-I	*Cx.* (*Mel.*) *theobaldi*	54330	54330	106799	98,29	1861	11816
PQ411	MG08-102	*Cx.* (*Mel.*) *dunni*	70736	70736	139413	98,54	2059	11808

TABLE III

Start codon and Stop codon of each protein coding gene.

Protein coding gene	Start codon	Stop codon
COX1	*	T__
COX2	ATG	T__
ATP8	ATT**	TAA
ATP6	ATG	TAA
COX3	ATG	TAA
ND3	ATT	TAA
ND5	ATC	TAA
ND4	ATG	TAA
ND4L	ATG	TAA
ND6	ATA	TAA
CYTB	ATG	TAA
ND1	ATA	TAA

*As COX1 was not sequenced in the beginning, this information was not possible; **ATC to *Culex ensiformis* (PQ405 and PQ407).

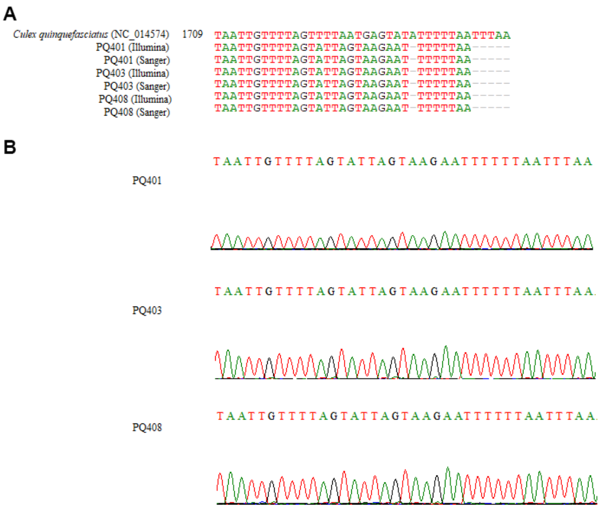

Fig. 1: schematic diagram showing the alignment of a *ND5* region. (A) *ND5* alignment of *Culex quinquefasciatus* (NC_014574) with three other samples from this study, considering the sequences obtained by Illumina and Sanger sequencing. (B) Electropherograms corresponding to the region aligned in (A), from three samples from this study.

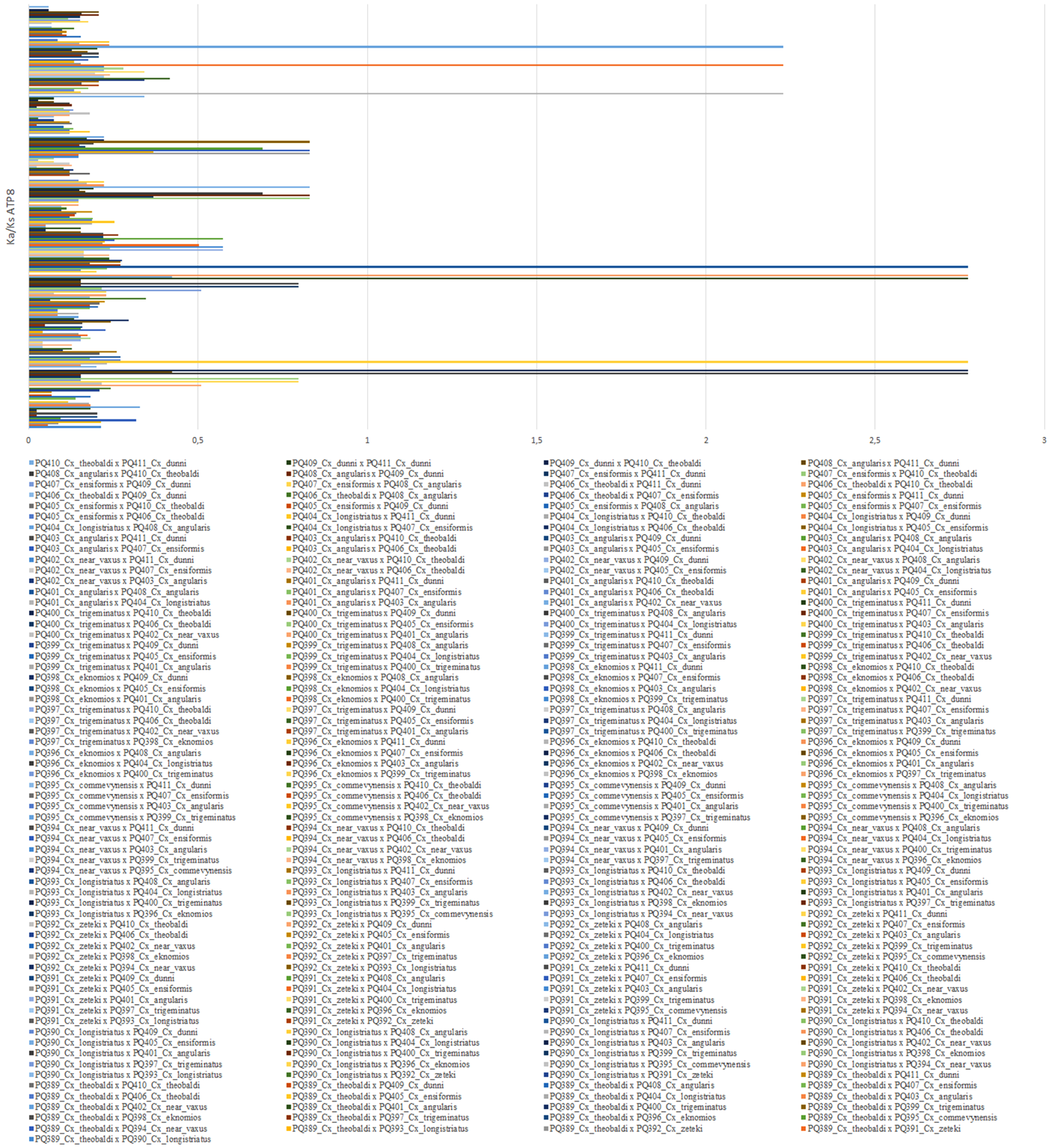

Fig. 2: Ka/Ks values of the *ATP8* gene of *Culex* (*Mel.*) species.

TABLE IV

Relative synonymous codon usage (RSCU) values of the mitochondrial partial genome of species of *Culex* (*Mel.*)

AA	codon	RSCU Cx_near_ commevynensis	RSCU Cx_dunni	RSCU Cx_eknomios	RSCU Cx_ensiformis	RSCU Cx_theobaldi	RSCU Cx_trigeminatus	RSCU Cx_longistriatus	RSCU Cx_angularis	RSCU Cx_near_vaxusPQ394	RSCU Cx_near_vaxusPQ402	RSCU_Cx_zeteki
Ala	GCU	2.08	2.32	2.33	2.21	2.14	1.98	2.36	2.39	2.25	2.39	2.35
Ala	GCC	0.61	0.42	0.33	0.55	0.5	0.76	0.29	0.18	0.46	0.25	0.38
Ala	GCA	1.09	1.12	1.26	1.11	1.17	1.13	1.26	1.3	1.08	1.22	1.13
Ala	GCG	0.22	0.13	0.08	0.13	0.19	0.14	0.09	0.13	0.21	0.13	0.13
Arg	CGU	0.37	0.37	0.96	0.26	0.52	0.59	0.84	0.81	0.83	0.37	0.4
Arg	CGC	0	0.07	0.15	0.04	0.07	0	0.2	0.15	0.08	0.15	0.15
Arg	CGA	2.96	3.3	2.44	3.19	2.94	2.89	2.77	2.59	2.64	2.96	2.91
Arg	CGG	0.67	0.26	0.44	0.52	0.47	0.52	0.2	0.44	0.45	0.52	0.55
Asn	AAU	1.68	1.76	1.93	1.67	1.75	1.66	1.87	1.83	1.85	1.85	1.69
Asn	AAC	0.32	0.24	0.07	0.33	0.25	0.34	0.13	0.17	0.15	0.15	0.31
Asp	GAU	1.43	1.67	1.72	1.57	1.69	1.57	1.65	1.64	1.66	1.75	1.64
Asp	GAC	0.57	0.33	0.28	0.43	0.31	0.43	0.35	0.36	0.34	0.25	0.36
Cys	UGU	1.76	1.95	1.72	1.71	1.87	1.68	1.85	1.78	1.82	1.76	1.71
Cys	UGC	0.24	0.05	0.28	0.29	0.13	0.32	0.15	0.22	0.18	0.24	0.29
Gln	CAA	1.94	1.83	1.83	1.79	1.9	1.76	1.91	1.92	1.83	1.94	1.86
Gln	CAG	0.06	0.17	0.17	0.21	0.1	0.24	0.09	0.08	0.17	0.06	0.14
Glu	GAA	1.74	1.81	1.78	1.76	1.8	1.66	1.85	1.8	1.71	1.86	1.74
Glu	GAG	0.26	0.19	0.22	0.24	0.2	0.34	0.15	0.2	0.29	0.14	0.26
Gly	GGU	0.52	0.71	0.76	0.71	0.67	0.44	0.68	0.72	0.63	0.79	0.63
Gly	GGC	0.16	0.08	0.14	0.15	0.05	0.06	0.09	0.1	0.2	0.08	0.14
Gly	GGA	2.03	2.24	2.34	1.8	2.24	2.22	2.47	2.42	2.13	2.29	2.14
Gly	GGG	1.29	0.97	0.77	1.34	1.04	1.28	0.76	0.76	1.04	0.83	1.09
His	CAU	1.32	1.62	1.53	1.45	1.41	1.4	1.63	1.51	1.54	1.69	1.58
His	CAC	0.68	0.38	0.47	0.55	0.59	0.6	0.37	0.49	0.46	0.31	0.42
Ile	AUU	1.9	1.92	1.93	1.9	1.95	1.87	1.94	1.94	1.88	1.93	1.89
Ile	AUC	0.1	0.08	0.07	0.1	0.05	0.13	0.06	0.06	0.12	0.07	0.11
Leu1	UUA	4.83	4.76	5.01	4.77	5.09	4.6	5.09	4.99	4.99	5.14	4.78
Leu1	UUG	0.28	0.36	0.27	0.31	0.29	0.45	0.21	0.25	0.29	0.18	0.32
Leu2	CUU	0.48	0.45	0.37	0.43	0.28	0.51	0.37	0.39	0.38	0.34	0.45
Leu2	CUC	0.01	0.01	0.03	0.04	0.01	0.04	0.02	0.01	0.05	0.01	0.05
Leu2	CUA	0.36	0.41	0.32	0.43	0.3	0.38	0.32	0.36	0.25	0.31	0.35
Leu2	CUG	0.05	0.02	0	0.02	0.02	0.02	0	0	0.03	0.01	0.05
Lys	AAA	1.51	1.54	1.85	1.52	1.5	1.65	1.76	1.66	1.88	1.85	1.48
Lys	AAG	0.49	0.46	0.15	0.48	0.5	0.35	0.24	0.34	0.12	0.15	0.52
Met	AUA	1.79	1.83	1.82	1.82	1.76	1.73	1.85	1.78	1.82	1.86	1.77
Met	AUG	0.21	0.17	0.18	0.18	0.24	0.27	0.15	0.22	0.18	0.14	0.24
Phe	UUU	1.83	1.79	1.88	1.75	1.83	1.83	1.87	1.84	1.96	1.86	1.8

AA	codon	RSCU Cx_near_ commevynensis	RSCU Cx_dunni	RSCU Cx_eknomios	RSCU Cx_ensiformis	RSCU Cx_theobaldi	RSCU Cx_trigeminatus	RSCU Cx_longistriatus	RSCU Cx_angularis	RSCU Cx_near_vaxusPQ394	RSCU Cx_near_vaxusPQ402	RSCU_Cx_zeteki
Phe	UUC	0.17	0.21	0.12	0.25	0.17	0.17	0.13	0.16	0.07	0.14	0.2
Pro	CCU	2.07	2.26	2.47	2.15	2.26	1.8	2.51	2.47	2.18	2.28	2.33
Pro	CCC	0.77	0.53	0.43	0.64	0.52	0.88	0.32	0.36	0.56	0.56	0.46
Pro	CCA	1.13	1.09	1.03	1.14	1.07	1.25	1.13	1.13	1.16	1.12	1.17
Pro	CCG	0.03	0.12	0.07	0.07	0.15	0.08	0.03	0.03	0.1	0.03	0.03
Ser1	AGU	1.45	1.48	1.53	1.53	1.85	1.56	1.61	1.63	1.37	1.63	1.61
Ser1	AGC	0.26	0.17	0.17	0.13	0.15	0.18	0.06	0.11	0.17	0.03	0.13
Ser1	AGA	1.45	1.47	1.36	1.49	1.08	1.41	1.37	1.35	1.49	1.47	1.4
Ser1	AGG	0	0.03	0.03	0	0	0.03	0	0	0	0	0.01
Ser2	UCU	3.4	3.41	3.54	3.52	3.14	3.39	3.53	3.36	3.34	3.13	3.41
Ser2	UCC	0.28	0.28	0.14	0.24	0.22	0.2	0.08	0.15	0.34	0.14	0.25
Ser2	UCA	1.11	1.14	1.2	1.04	1.55	1.23	1.34	1.37	1.29	1.61	1.15
Ser2	UCG	0.06	0.03	0.03	0.07	0.01	0	0	0.04	0	0	0.03
Thr	ACU	2.09	2.04	1.88	2.13	2.07	2.24	2.07	2.05	1.91	1.94	2.04
Thr	ACC	0.29	0.31	0.13	0.26	0.06	0.22	0.14	0.08	0.15	0.24	0.36
Thr	ACA	1.47	1.59	1.9	1.53	1.76	1.52	1.79	1.84	1.83	1.75	1.55
Thr	ACG	0.16	0.06	0.09	0.08	0.1	0.02	0.01	0.02	0.11	0.07	0.04
Trp	UGA	1.91	1.93	1.89	1.88	1.89	1.93	1.81	1.84	1.71	1.89	1.83
Trp	UGG	0.09	0.07	0.11	0.12	0.11	0.07	0.19	0.16	0.29	0.11	0.17
Tyr	UAU	1.79	1.69	1.8	1.66	1.71	1.72	1.81	1.74	1.74	1.79	1.71
Tyr	UAC	0.21	0.31	0.2	0.34	0.29	0.82	0.19	0.26	0.26	0.21	0.29
Val	GUU	1.63	1.82	1.85	1.77	2	1.79	1.97	1.89	1.68	1.98	1.78
Val	GUC	0.11	0.16	0.14	0.18	0.11	0.25	0.02	0.02	0.2	0.12	0.1
Val	GUA	2.01	1.85	1.92	1.74	1.8	1.81	1.86	1.92	1.95	1.69	1.88
Val	GUG	0.25	0.17	0.09	0.31	0.09	0.15	0.14	0.16	0.17	0.22	0.24

TABLE V

Nucleotide diversity of mitochondrial partial genome of *Culex* (*Mel.*)

Window	Midpoint	Pi
1-200	100	0,05372
26-225	125	0,06225
51-250	150	0,06413
76-275	175	0,05933
101-300	200	0,07322
126-325	225	0,0763
151-350	250	0,08279
176-375	275	0,09128
201-400	300	0,10727
226-425	325	0,11348
251-450	350	0,10915
276-475	375	0,10868
301-500	400	0,09854
326-525	425	0,10047
351-550	450	0,10014
376-575	475	0,1101
401-600	500	0,09958
426-625	525	0,0886
451-650	550	0,09069
476-675	575	0,09053
501-700	600	0,0946
526-725	625	0,08737
551-750	650	0,07885
576-775	675	0,06613
601-800	700	0,06385
626-825	725	0,06571
651-850	750	0,06668
676-875	775	0,06599
701-900	800	0,06757
726-925	825	0,07506
751-950	850	0,08291
776-975	875	0,08421
801-1000	900	0,08611
826-1025	925	0,08498
851-1050	950	0,08255
876-1075	975	0,08893
901-1100	1000	0,08814
926-1125	1025	0,09358
951-1150	1050	0,08694
976-1175	1075	0,09451
1001-1200	1100	0,10213
1026-1225	1125	0,10071
1051-1250	1150	0,10443
1076-1275	1175	0,10225
1101-1300	1200	0,10393
1126-1325	1225	0,09769
1151-1350	1250	0,10166
1176-1375	1275	0,09164
1201-1400	1300	0,07711
1226-1425	1325	0,08213
1251-1450	1350	0,07621
1276-1475	1375	0,07773
1301-1500	1400	0,07233
1326-1525	1425	0,06261
1351-1550	1450	0,05221
1376-1576	1475	0,05391
1401-1605	1500	0,05146
1426-1630	1525	0,05014
1451-1655	1550	0,04559
1476-1680	1576	0,03941
1501-1705	1605	0,03759
1526-1730	1630	0,04585
1551-1755	1655	0,05577
1577-1780	1680	0,05429
1606-1805	1705	0,06267
1631-1830	1730	0,06389
1656-1855	1755	0,06854
1681-1880	1780	0,07735
1706-1905	1805	0,08156
1731-1930	1830	0,08126
1756-1955	1855	0,07545
1781-1980	1880	0,08688
1806-2005	1905	0,09105
1831-2030	1930	0,09166
1856-2055	1955	0,09435
1881-2080	1980	0,0935
1906-2105	2005	0,08996
1931-2130	2030	0,09405
1956-2155	2055	0,09435
1981-2180	2080	0,09
2006-2205	2105	0,08208
2031-2230	2130	0,0751
2056-2255	2155	0,06808
2081-2280	2180	0,0553
2106-2305	2205	0,04676
2131-2330	2230	0,03899
2156-2361	2255	0,04217
2181-2386	2280	0,03231
2206-2412	2305	0,02812
2231-2437	2330	0,02409
2256-2462	2361	0,0237
2281-2487	2386	0,03753
2306-2512	2412	0,04077
2331-2537	2437	0,05425
2362-2562	2462	0,06204
2387-2587	2487	0,06721
2413-2612	2512	0,07955
2438-2637	2537	0,08883
2463-2662	2562	0,09725
2488-2687	2587	0,09209
2513-2712	2612	0,09773
2538-2737	2637	0,08482
2563-2762	2662	0,0813
2588-2787	2687	0,08891
2613-2812	2712	0,08761
2638-2837	2737	0,08737
2663-2862	2762	0,08832
2688-2887	2787	0,08905
2713-2912	2812	0,09676
2738-2937	2837	0,09621

↳

Window	Midpoint	Pi
2763-2962	2862	0,09257
2788-2987	2887	0,09296
2813-3012	2912	0,09804
2838-3037	2937	0,09516
2863-3062	2962	0,09091
2888-3087	2987	0,09162
2913-3112	3012	0,08405
2938-3138	3037	0,09411
2963-3163	3062	0,09644
2988-3188	3087	0,08747
3013-3213	3112	0,0804
3038-3238	3138	0,07765
3063-3263	3163	0,07292
3088-3288	3188	0,07168
3113-3313	3213	0,07239
3139-3338	3238	0,06895
3164-3363	3263	0,07243
3189-3388	3288	0,07907
3214-3413	3313	0,08134
3239-3438	3338	0,08018
3264-3463	3363	0,08692
3289-3488	3388	0,09451
3314-3513	3413	0,09567
3339-3538	3438	0,09324
3364-3563	3463	0,0865
3389-3588	3488	0,08783
3414-3613	3513	0,07976
3439-3638	3538	0,09636
3464-3663	3563	0,0916
3489-3688	3588	0,08405
3514-3713	3613	0,08458
3539-3738	3638	0,08277
3564-3763	3663	0,08731
3589-3788	3688	0,08646
3614-3813	3713	0,09026
3639-3838	3738	0,08174
3664-3863	3763	0,08721
3689-3888	3788	0,09314
3714-3913	3813	0,08856
3739-3938	3838	0,09542
3764-3963	3863	0,09417
3789-3988	3888	0,09136
3814-4013	3913	0,09061
3839-4038	3938	0,08063
3864-4063	3963	0,06903
3889-4089	3988	0,05937
3914-4115	4013	0,05403
3939-4140	4038	0,05374
3964-4165	4063	0,05385
3989-4190	4089	0,05075
4014-4215	4115	0,05051
4039-4240	4140	0,05909
4064-4265	4165	0,06749
4090-4290	4190	0,07348
4116-4315	4215	0,07486
4141-4340	4240	0,06372
4166-4365	4265	0,06164

Window	Midpoint	Pi
4191-4390	4290	0,06411
4216-4415	4315	0,06245
4241-4440	4340	0,0666
4266-4465	4365	0,07462
4291-4490	4390	0,06717
4316-4515	4415	0,06749
4341-4553	4440	0,07132
4366-4578	4465	0,06632
4391-4603	4490	0,05595
4416-4636	4515	0,05231
4441-4661	4553	0,03923
4466-4688	4578	0,02775
4491-4713	4603	0,02435
4516-4738	4636	0,02115
4554-4763	4661	0,01684
4579-4789	4688	0,01846
4604-4817	4713	0,01889
4637-4842	4738	0,0236
4662-4867	4763	0,03198
4689-4901	4789	0,02996
4714-4933	4817	0,0384
4739-4958	4842	0,04591
4764-4983	4867	0,04294
4790-5008	4901	0,04739
4818-5034	4933	0,04976
4843-5059	4958	0,05356
4868-5084	4983	0,04931
4902-5109	5008	0,0546
4934-5134	5034	0,06067
4959-5159	5059	0,06478
4984-5184	5084	0,09306
5009-5209	5109	0,09781
5035-5234	5134	0,10093
5060-5259	5159	0,11166
5085-5284	5184	0,11318
5110-5309	5209	0,11484
5135-5334	5234	0,10682
5160-5359	5259	0,11101
5185-5384	5284	0,08858
5210-5409	5309	0,08791
5235-5434	5334	0,08486
5260-5459	5359	0,07308
5285-5484	5384	0,0736
5310-5509	5409	0,06717
5335-5534	5434	0,06504
5360-5559	5459	0,05642
5385-5584	5484	0,05779
5410-5609	5509	0,05751
5435-5634	5534	0,06221
5460-5659	5559	0,06532
5485-5684	5584	0,06453
5510-5709	5609	0,07049
5535-5734	5634	0,0781
5560-5759	5659	0,08032
5585-5784	5684	0,0849
5610-5809	5709	0,08565
5635-5834	5734	0,07927

Window	Midpoint	Pi
5660-5859	5759	0,07211
5685-5884	5784	0,07923
5710-5909	5809	0,08024
5735-5934	5834	0,082
5760-5959	5859	0,07893
5785-5984	5884	0,08186
5810-6009	5909	0,07996
5835-6034	5934	0,09759
5860-6059	5959	0,09743
5885-6084	5984	0,09791
5910-6109	6009	0,10354
5935-6134	6034	0,09551
5960-6159	6059	0,09644
5985-6184	6084	0,08595
6010-6209	6109	0,08698
6035-6234	6134	0,07804
6060-6259	6159	0,08289
6085-6284	6184	0,07219
6110-6309	6209	0,0736
6135-6334	6234	0,08053
6160-6359	6259	0,08099
6185-6384	6284	0,08411
6210-6409	6309	0,08421
6235-6434	6334	0,07903
6260-6459	6359	0,07352
6285-6484	6384	0,07528
6310-6509	6409	0,06522
6335-6534	6434	0,06646
6360-6559	6459	0,06549
6385-6584	6484	0,07126
6410-6609	6509	0,07105
6435-6634	6534	0,06842
6460-6659	6559	0,06822
6485-6687	6584	0,06455
6510-6714	6609	0,05757
6535-6739	6634	0,04417
6560-6764	6659	0,04306
6585-6789	6687	0,038
6610-6814	6714	0,04451
6635-6839	6739	0,05087
6660-6864	6764	0,05601
6688-6889	6789	0,06617
6715-6914	6814	0,07
6740-6939	6839	0,07996
6765-6964	6864	0,08644
6790-6989	6889	0,08625
6815-7014	6914	0,07725
6840-7039	6939	0,08334
6865-7064	6964	0,08514
6890-7089	6989	0,08767
6915-7114	7014	0,09233
6940-7139	7039	0,08951
6965-7164	7064	0,08986
6990-7189	7089	0,0915
7015-7214	7114	0,09383
7040-7239	7139	0,0886
7065-7264	7164	0,08899

Window	Midpoint	Pi
7090-7289	7189	0,08852
7115-7314	7214	0,08812
7140-7339	7239	0,09239
7165-7364	7264	0,0935
7190-7389	7289	0,09796
7215-7414	7314	0,09026
7240-7439	7339	0,09547
7265-7464	7364	0,09581
7290-7489	7389	0,09162
7315-7514	7414	0,09099
7340-7539	7439	0,09314
7365-7564	7464	0,09302
7390-7589	7489	0,09453
7415-7614	7514	0,1013
7440-7639	7539	0,09727
7465-7664	7564	0,09824
7490-7689	7589	0,10119
7515-7714	7614	0,10868
7540-7739	7639	0,09634
7565-7764	7664	0,09314
7590-7789	7689	0,0887
7615-7814	7714	0,07901
7640-7839	7739	0,08824
7665-7864	7764	0,08796
7690-7889	7789	0,08741
7715-7914	7814	0,08059
7740-7939	7839	0,07972
7765-7964	7864	0,07864
7790-7989	7889	0,07148
7815-8014	7914	0,07061
7840-8039	7939	0,05939
7865-8064	7964	0,05115
7890-8089	7989	0,04457
7915-8114	8014	0,04553
7940-8139	8039	0,05599
7965-8164	8064	0,05506
7990-8189	8089	0,05676
8015-8214	8114	0,06055
8040-8239	8139	0,05929
8065-8264	8164	0,06304
8090-8289	8189	0,06755
8115-8314	8214	0,06356
8140-8339	8239	0,05547
8165-8365	8264	0,05472
8190-8390	8289	0,05121
8215-8420	8314	0,04623
8240-8445	8339	0,04115
8265-8470	8365	0,03739
8290-8496	8390	0,02791
8315-8521	8420	0,0331
8340-8546	8445	0,03547
8366-8571	8470	0,03818
8391-8596	8496	0,05125
8421-8621	8521	0,05909
8446-8646	8546	0,06457
8471-8671	8571	0,06516
8497-8696	8596	0,07294

Window	Midpoint	Pi
8522-8721	8621	0,06874
8547-8746	8646	0,07055
8572-8771	8671	0,08658
8597-8796	8696	0,0934
8622-8821	8721	0,11026
8647-8846	8746	0,11717
8672-8871	8771	0,12085
8697-8896	8796	0,1154
8722-8921	8821	0,12055
8747-8946	8846	0,1204
8772-8971	8871	0,09913
8797-8996	8896	0,07953
8822-9025	8921	0,07176
8847-9050	8946	0,0636
8872-9075	8971	0,06379
8897-9100	8996	0,07874
8922-9125	9025	0,07344
8947-9150	9050	0,07318
8972-9175	9075	0,07423
8997-9200	9100	0,08543
9026-9225	9125	0,0769
9051-9250	9150	0,08257
9076-9275	9175	0,08901
9101-9300	9200	0,08051
9126-9325	9225	0,0937
9151-9350	9250	0,09328
9176-9375	9275	0,10563
9201-9400	9300	0,09929
9226-9425	9325	0,10291
9251-9450	9350	0,09822
9276-9475	9375	0,0947
9301-9500	9400	0,09696
9326-9525	9425	0,08257
9351-9550	9450	0,08474
9376-9575	9475	0,0736
9401-9600	9500	0,07789
9426-9625	9525	0,07083
9451-9650	9550	0,07634
9476-9675	9575	0,07528
9501-9700	9600	0,08225
9526-9725	9625	0,09101
9551-9750	9650	0,08958
9576-9775	9675	0,08682
9601-9800	9700	0,08409
9626-9825	9725	0,09785
9651-9850	9750	0,09731
9676-9875	9775	0,09279
9701-9900	9800	0,08415
9726-9926	9825	0,08028
9751-9951	9850	0,08275
9776-9976	9875	0,09223
9801-10001	9900	0,09364
9826-10026	9926	0,08836
9851-10051	9951	0,08893
9876-10076	9976	0,08947
9901-10101	10001	0,09296
9927-10126	10026	0,09474

Window	Midpoint	Pi
9952-10151	10051	0,09002
9977-10177	10076	0,078
10002-10202	10101	0,06962
10027-10246	10126	0,06508
10052-10271	10151	0,05381
10077-10296	10177	0,06148
10102-10321	10202	0,04901
10127-10346	10246	0,04273
10152-10371	10271	0,04528
10178-10396	10296	0,05455
10203-10421	10321	0,05883
10247-10446	10346	0,05271
10272-10472	10371	0,05638
10297-10497	10396	0,0501
10322-10522	10421	0,05488
10347-10547	10446	0,05281
10372-10572	10472	0,04895
10397-10597	10497	0,05162
10422-10622	10522	0,05787
10447-10647	10547	0,06344
10473-10672	10572	0,06427
10498-10697	10597	0,06593
10523-10722	10622	0,0667
10548-10747	10647	0,06621
10573-10772	10672	0,06581
10598-10797	10697	0,06662
10623-10822	10722	0,06739
10648-10847	10747	0,0766
10673-10872	10772	0,0768
10698-10897	10797	0,07451
10723-10922	10822	0,07528
10748-10947	10847	0,07773
10773-10972	10872	0,07745
10798-10997	10897	0,06403
10823-11022	10922	0,06217
10848-11047	10947	0,05308
10873-11072	10972	0,06581
10898-11097	10997	0,06935
10923-11122	11022	0,0765
10948-11147	11047	0,08194
10973-11172	11072	0,08407
10998-11197	11097	0,08605
11023-11225	11122	0,07439
11048-11253	11147	0,07166
11073-11298	11172	0,06105
11098-11327	11197	0,06289
11123-11352	11225	0,05071
11148-11377	11253	0,04486
11173-11402	11298	0,03846
11198-11427	11327	0,03522
11226-11452	11352	0,03684
11254-11477	11377	0,03111
11299-11502	11402	0,02298
11328-11527	11427	0,01168
11353-11552	11452	0,00911
11378-11577	11477	0,00407
11403-11602	11502	0,01134

Window	Midpoint	Pi
11428-11627	11527	0,01391
11453-11652	11552	0,01474
11478-11677	11577	0,01751
11503-11702	11602	0,02209
11528-11727	11627	0,02209
11553-11752	11652	0,02209
11578-11777	11677	0,02209
11603-11802	11702	0,01719
11628-11827	11727	0,01506
11653-11852	11752	0,01482
11678-11877	11777	0,01162
11703-11890	11796	0,00622
